# Treatment of a compound calcaneus fracture Sanders IV with an external circular fixator and calcaneal osteotomy

**DOI:** 10.1016/j.tcr.2023.100850

**Published:** 2023-05-20

**Authors:** Ofer Heinig, Elia Feicht, Assil Mahamid, Roman Liberson, Claude Picard, Aharon Liberson

**Affiliations:** a Foot & Ankle Unite, Laniado University Hospital, Adelson Faculty of Health Sciences, Ariel University, Ariel, Israel; b Faculty of Health Sciences, Ben-Gurion University of the Negev, Beer-Sheva, Israel

**Keywords:** Calcaneal fracture, External fixation, Ilizarov technique, Compound

## Abstract

Compound Gustilo-type III intra-articular calcaneus fractures are challenging to treat. Anatomical reduction of the subtalar joint increases the chances of a better functional outcome and is traditionally achieved by an open reduction and plating. Conversely, ORIF is associated with a high risk of infection and even amputation. In our case study, we present the treatment of a Gustilo-type III intra-articular calcaneus fracture with a circular external fixator and a temporary antibiotic cement spacer for fracture reduction and stabilization. Active bio-glass was implanted to fill bone loss and to prevent infection. A closing-wedge calcaneal tuberosity osteotomy was used to facilitate wound closure. We paid special attention to reducing the posterior facet. The patient returned to work and full ambulation five months post-injury.

## Introduction

Open calcaneal fractures make up between 0.8 % and 10 % of all calcaneal fractures [1]. The treatment of these fractures is challenging and is associated with severe complications such as infection, osteomyelitis, or even amputation [2]. We present a case of an open Gustilo IIIb and Sanders IV calcaneus fracture that was treated with an external circular fixator, temporary antibiotic cement spacer to prevent infection, bioactive glass to fill bone loss, and a calcaneal tuberosity closing-wedge osteotomy with skin grafting for wound closure. This treatment resulted in full ambulation and return to work five months post-injury.

## Methods

The clinical records were acquired from the patient with informed consent after approval by the institutional ethics committee.

## Patient information

**Fig. 1:**
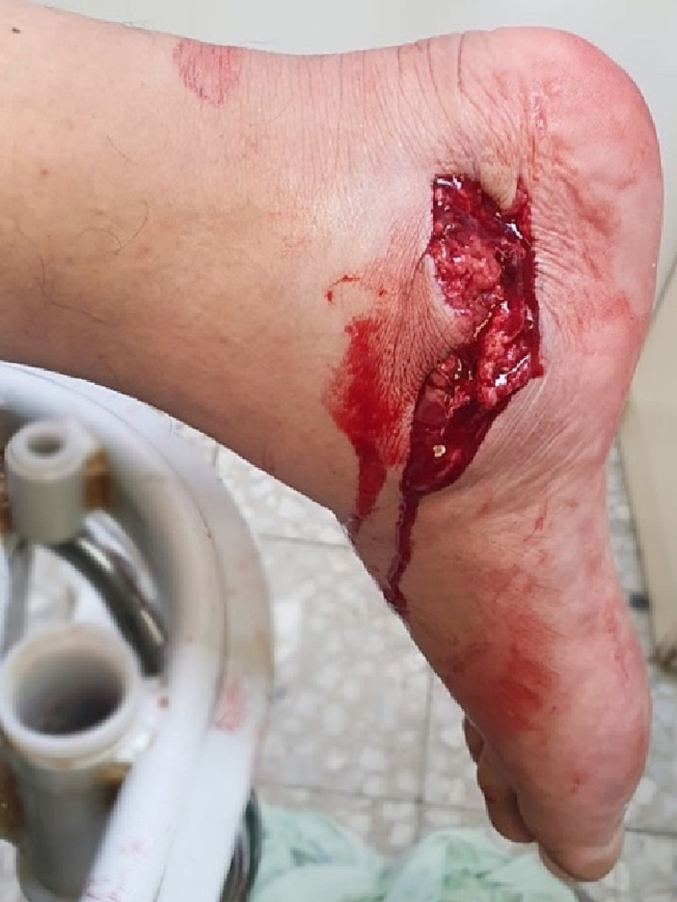
Medial hindfoot wound on presentation to ER.

A 45-year-old male construction worker was admitted to our Orthopedics Department, with an open wound on the medial side of his right heel (Fig. 1), foot swelling, pain, and medial ecchymosis. The patient fell from a 4-meter height and landed on his right heel.

Physical examination revealed an 8 ∗ 3 cm wound just distal to the medial malleolus with active capillary bleeding and exposure of the calcaneal bone. The patient was able to actively flex and extend his toes. No sensory defect was noted. The dorsalis pedis pulse was palpated.

**Fig. 2:**
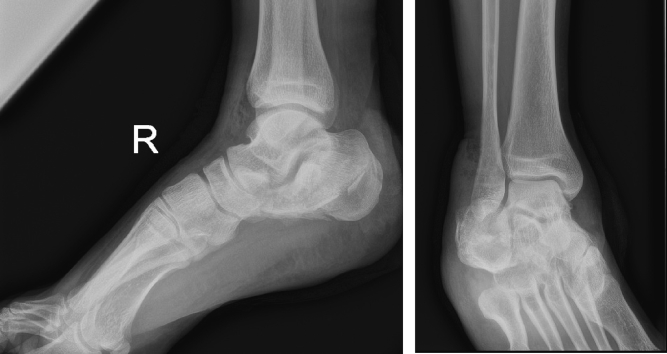
AP + lateral ankle x-ray on presentation to ER.

**Fig. 3:**
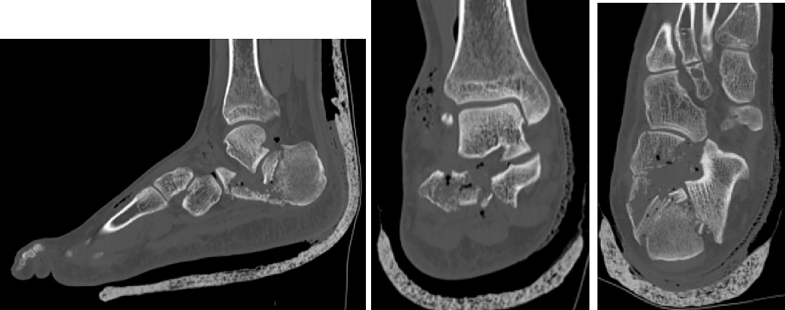
Sagittal, Coronal and Axial CT-scan after initial splinting in ER.

The X-ray (Fig. 2) showed a comminuted intra-articular calcaneal fracture with a depression of the posterior calcaneal facet and a Boehler angle of 8 degrees. A CT scan (Fig. 3) revealed a Sanders Type IV fracture with severe valgus deformity and bone impaction.

In the emergency department, the patient underwent wound irrigation with several liters of 0.9 % NaCl saline. He was administered tetanus prophylaxis and IV Cefazolin and Gentamycin antibiotics. The skin was approximated with simple sutures and a splint was applied.

## Surgical technique

**Fig. 4:**
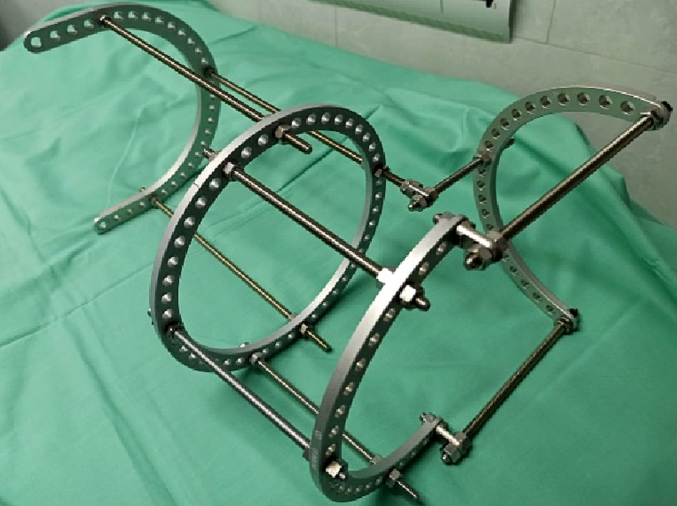
External circular fixator.

The patient was taken to the operating room and underwent irrigation and debridement open reduction and fixation with an external circular fixator (Fig. 4).

**Fig. 5:**
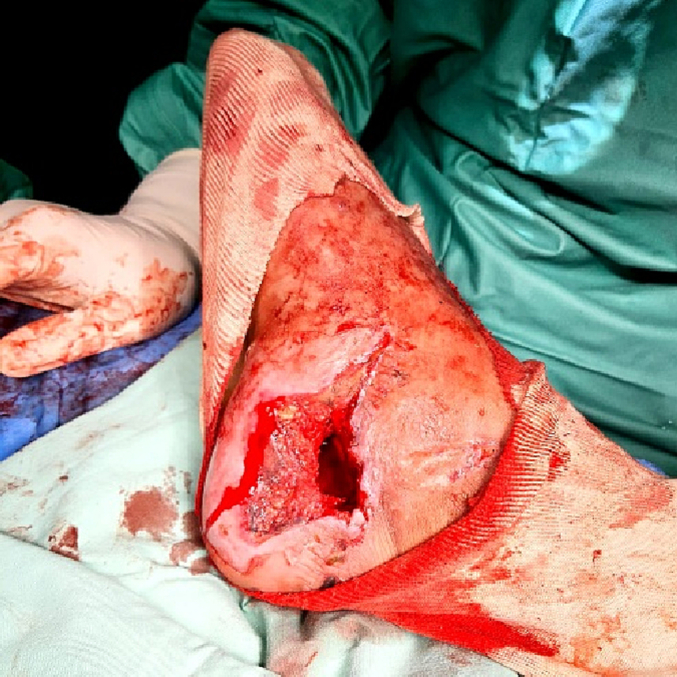
Initial irrigation and debridement in OR.

The skin stitches were opened, and exploration of the wound revealed a loss of soft tissue up to 10 cm in length, spanning from the calcaneal tuberosity up to the midfoot (Fig. 5).

Microbial cultures were taken and the wound was irrigated with additional Gentamycin solution and debrided.

**Fig. 6:**
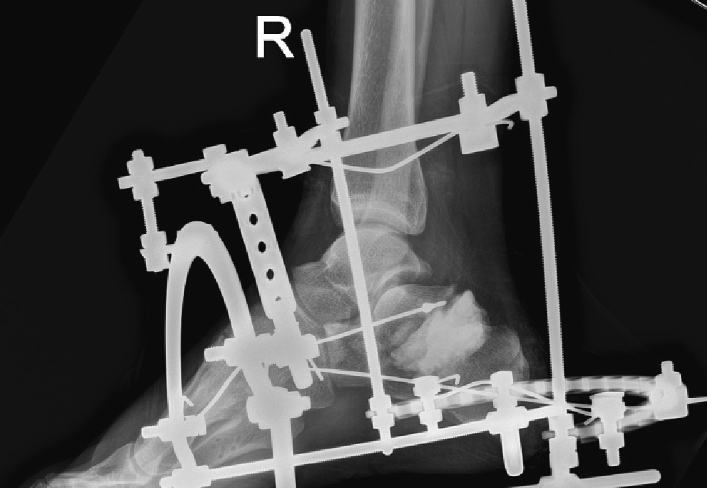
Post-operative lateral x-ray.

**Fig. 7:**
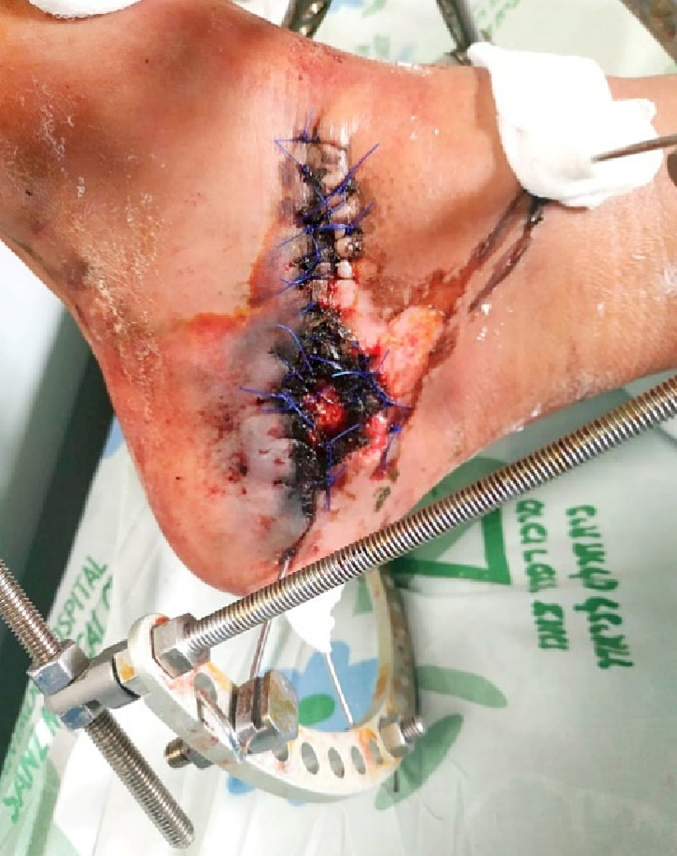
Signs of skin necrosis over medial hindfoot.

Wires were inserted into the calcaneal tuberosity, the bone fragment with the posterior facet, metatarsi 1–3, and the distal third of the tibia. The reduction was performed, the KW in the calcaneal tuberosity was advanced to the anterior process of the calcaneus and the KWs were fixated on the external circular fixator. The bone loss between the calcaneal tuberosity and the bone fragment holding the posterior facet was filled with PMMA Gentamycin cement (Fig. 6). The wound edges were approximated. The wound was treated with Prontosan dressings. Within a week the skin and subcutaneous tissue surrounding the wound started showing signs of necrosis (Fig. 7) and the patient underwent multiple debridements of the necrotic soft tissue and non-viable bone fragments.

The continued distraction of 1 mm per day was applied to the calcaneal tuberosity for 25 days to improve the calcaneal pitch while minimizing tension on the wound.

**Fig. 8:**
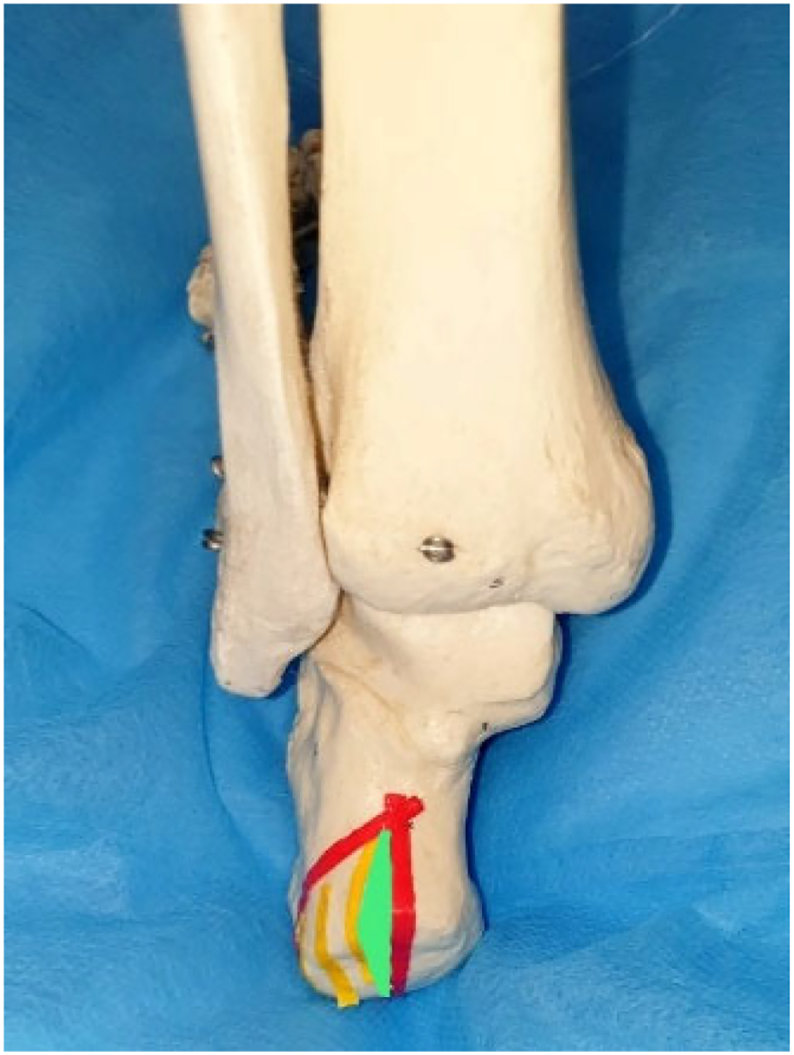
Pre-surgical planning of calcaneal closing-wedge osteotomy with partial excision of the middle calcaneal tuberosity. The green area represents the excised fragment. (For interpretation of the references to colour in this figure legend, the reader is referred to the web version of this article.)

**Fig. 9:**
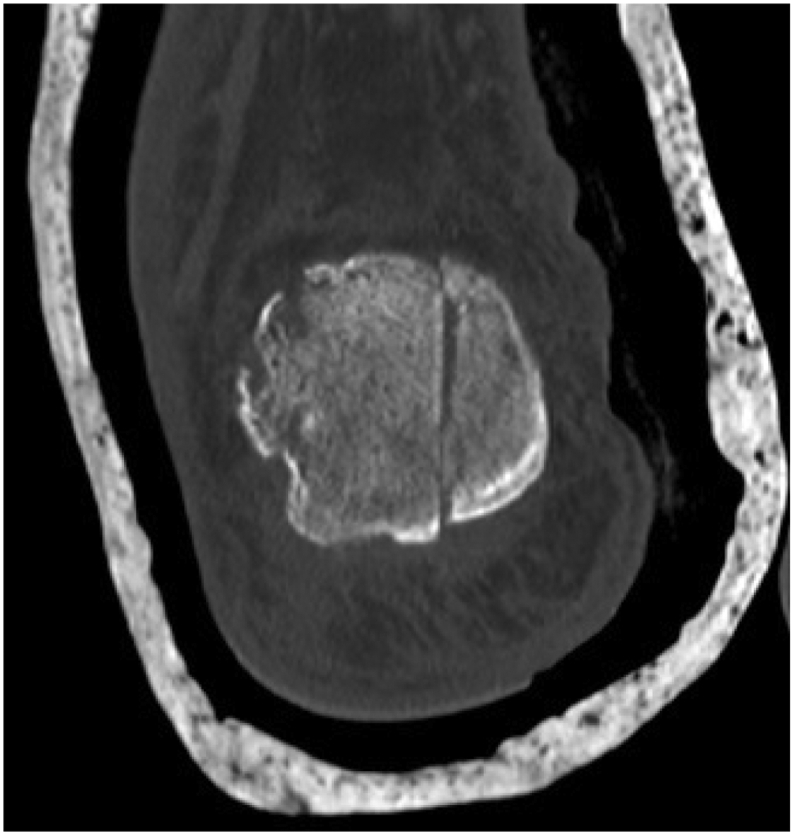
Post-operative coronal CT-slide showing the closing wedge calcaneal osteotomy.

**Fig. 10:**
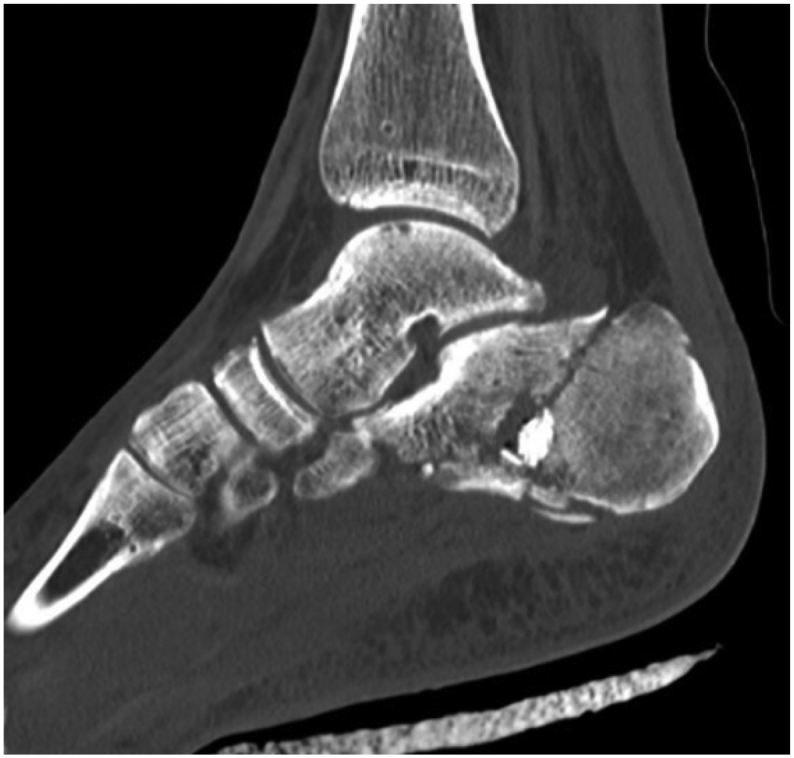
Post-operative sagittal CT slide showing the hyperdense active glass that was used to fill the bone defect.

One-month post-injury, to carry out wound closure, the patient underwent a medial calcaneal closing-wedge osteotomy with partial excision of the middle calcaneal tuberosity, while preserving the posterior facet of the subtalar joint (Fig. 8, Fig. 9, Fig. 10).

**Fig. 11:**
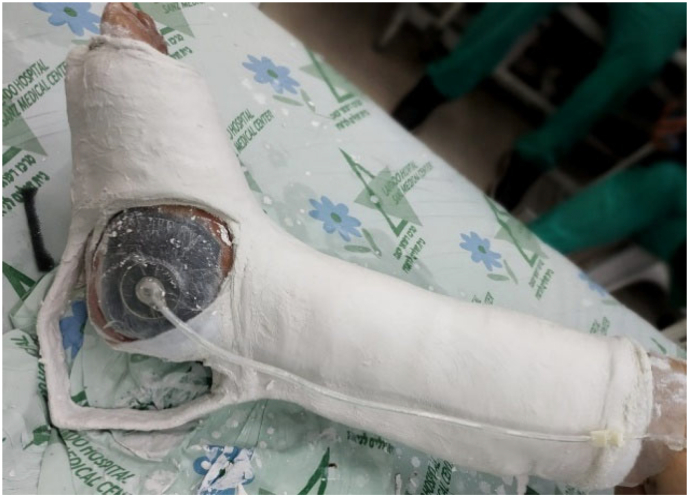
Cast with a modified Boehler attachment to allow for partial weight bearing and VAC treatment in preparation for skin graft.

**Fig. 12:**
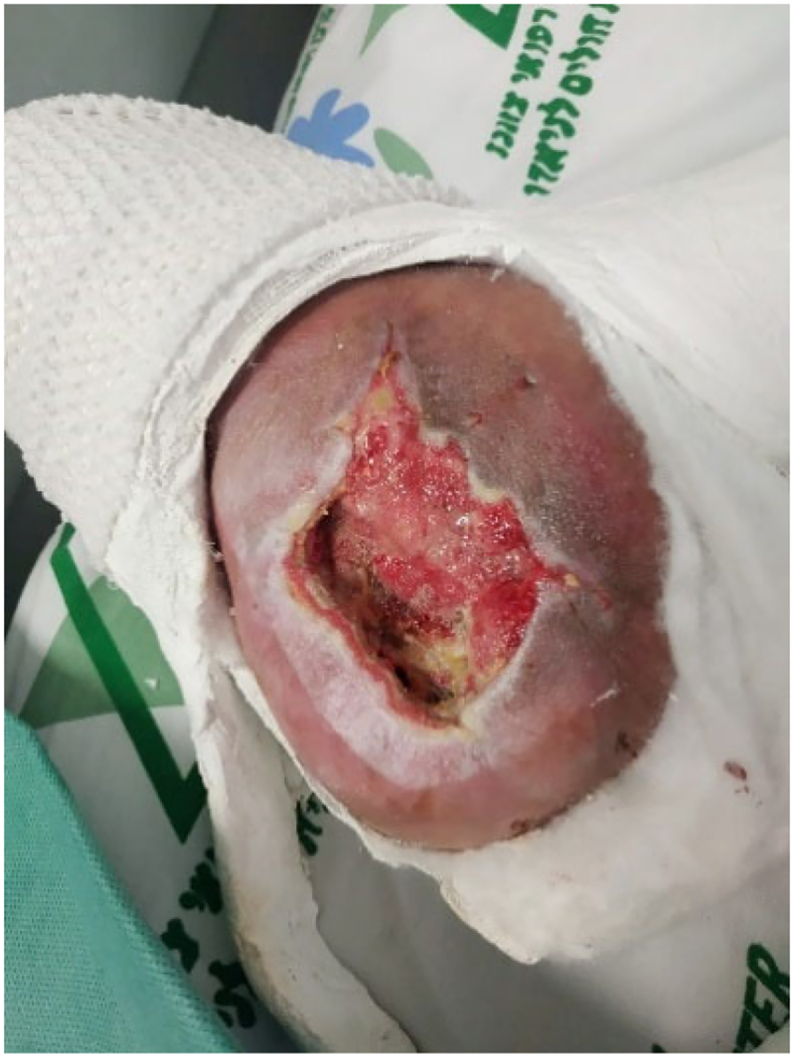
Wound after VAC treatment in preparation for skin graft.

**Fig. 13:**
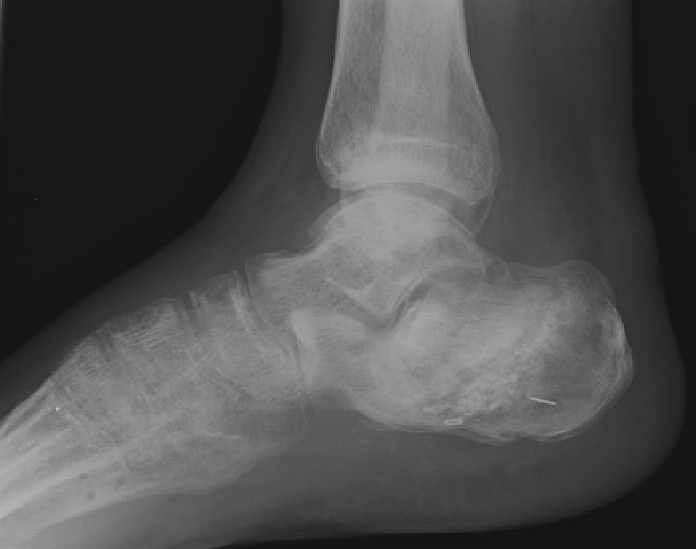
Five months post-injury lateral ankle x-ray.

The cement plug was replaced by a bioactive glass (bonealive). The external ring fixator was removed and the ankle stabilized in a cast with a modified Boehler attachment (Fig. 11) to allow for partial weight bearing and VAC treatment in preparation for skin graft (Fig. 12). The wound was then closed with a partial thickness skin graft from the thigh. Rehabilitation was initiated with weight-bearing as tolerated in a walking boot after full wound closure, and the patient returned to work and full ambulation after five months post-injury (Fig. 13).

## Discussion

The treatment of open calcaneus fractures is often complicated by infection. Deep tissue infection following open calcaneus fractures has been reported in up to 10–39 %, and eventual amputation in 8.0–14 % [3,4,5]. The prognosis is dependent on the Gustilo type. A retrospective study investigating 43 open calcaneus fractures showed that Gustilo type-I fractures treated with ORIF or with primary fusion had a good-to-excellent short-term result with no major complications, whereas Gustilo type-III fractures treated with early internal fixation have been associated with high rates of osteomyelitis and subsequent amputation [6]. Even in closed calcaneus fractures treated with ORIF deep wound infection has been reported to be as high as 13.6 % [5].

Anatomic reduction and fixation are thought to provide the best outcome in terms of the earliest possible restoration of function, patient satisfaction, and minimization of post-traumatic arthritis [8]. Functional and radiographic outcomes similar to those of ORIF have been reported using minimal open reduction and fixation with a circular external fixator [9,10,11].

Concerning our patient, we tried to achieve fracture reduction by open manipulation of the bone fragments through the existing medial wound. For fixation, we used a circular external fixator as a definite treatment to minimize further vascular damage and hence decrease the chances of deep tissue infection. The temporary antibiotic cement spacer helped us to stabilize the posterior facet, which has also been shown to decrease the risk for infection when used to fill bone defects in open fractures [12]. The cement spacer was replaced by bioactive glass putty to fill bone loss because of its antimicrobial and antibiofilm activity [13] and its bone-bonding properties [14]. Thinning of the calcaneal tuberosity allowed us to close the wound using a partial-thickness skin graft rather than having to utilize a free flap.

## Conclusions

The four key points we have drawn from our case study are as follows:

1. In compound calcaneus fractures, copious irrigation and debridement of the wound should be carried out and intra-venous antibiotic coverage should be administered as soon as possible.
2. Antibiotic cement spacer and consequent bioactive glass putty placement can help in maintaining fracture reduction and preventing infection.
3. A closing-wedge osteotomy of the calcaneal tuberosity can assist in wound closure and avert the use of a free flap
4. Good anatomical reduction and fixation can be achieved with an external circular fixator.
5. Both we and the patient were lucky to avoid nerve injury in this complicated area.

## Declaration of competing interest

The authors declare that they have no known competing financial interests or personal relationships that could have appeared to influence the work reported in this paper.
